# Staging of Liver Fibrosis Based on Energy Valley Optimization Multiple Stacking (EVO-MS) Model

**DOI:** 10.3390/bioengineering11050485

**Published:** 2024-05-13

**Authors:** Xuejun Zhang, Shengxiang Chen, Pengfei Zhang, Chun Wang, Qibo Wang, Xiangrong Zhou

**Affiliations:** 1 School of Computer, Electronics and Information, Guangxi University, Nanning 530004, China; xjzhang@gxu.edu.cn (X.Z.); 2107210341@st.gxu.edu.cn (P.Z.); 2107210346@st.gxu.edu.cn (C.W.);; 2 Guangxi Key Laboratory of Multimedia Communications and Network Technology, Guangxi University, Nanning 530004, China; 3 Department of Electrical, Electronic and Computer Engineering, Gifu University, Gifu 501-1193, Japan; zhou.xiangrong.n6@f.gifu-u.ac.jp

**Keywords:** ensemble learning, energy valley optimization algorithm, liver fibrosis, computer-aided diagnosis, image processing

## Abstract

Currently, staging the degree of liver fibrosis predominantly relies on liver biopsy, a method fraught with potential risks, such as bleeding and infection. With the rapid development of medical imaging devices, quantification of liver fibrosis through image processing technology has become feasible. Stacking technology is one of the effective ensemble techniques for potential usage, but precise tuning to find the optimal configuration manually is challenging. Therefore, this paper proposes a novel EVO-MS model—a multiple stacking ensemble learning model optimized by the energy valley optimization (EVO) algorithm to select most informatic features for fibrosis quantification. Liver contours are profiled from 415 biopsied proven CT cases, from which 10 shape features are calculated and inputted into a Support Vector Machine (SVM) classifier to generate the accurate predictions, then the EVO algorithm is applied to find the optimal parameter combination to fuse six base models: K-Nearest Neighbors (KNNs), Decision Tree (DT), Naive Bayes (NB), Extreme Gradient Boosting (XGB), Gradient Boosting Decision Tree (GBDT), and Random Forest (RF), to create a well-performing ensemble model. Experimental results indicate that selecting 3–5 feature parameters yields satisfactory results in classification, with features such as the contour roundness non-uniformity (Rmax), maximum peak height of contour (Rp), and maximum valley depth of contour (Rm) significantly influencing classification accuracy. The improved EVO algorithm, combined with a multiple stacking model, achieves an accuracy of 0.864, a precision of 0.813, a sensitivity of 0.912, a specificity of 0.824, and an F1-score of 0.860, which demonstrates the effectiveness of our EVO-MS model in staging the degree of liver fibrosis.

## 1. Introduction

Liver fibrosis is a common hepatic disease characterized by the abnormal proliferation and deposition of collagen fibers and other extracellular matrix components within the liver, resulting from chronic liver injury [1,2]. This pathological repair response is a critical step in the progression of various chronic liver diseases towards cirrhosis. The process is associated not only with chronic viral hepatitis, such as hepatitis B and C, but also with the incidence of fibrosis due to non-alcoholic fatty liver disease (NAFLD) and autoimmune liver diseases, which have also been increasing in recent years. Early diagnosis and accurate staging of liver fibrosis are of significant importance for treatment and prognosis. Traditional diagnostic methods for liver fibrosis primarily rely on liver tissue examination, namely, liver biopsy. Liver biopsy is considered the gold standard for diagnosing liver fibrosis [3,4,5]; however, its invasive nature, high cost, and associated risks limit its widespread clinical application. Therefore, there is an urgent need for a non-invasive and convenient method for diagnosing liver fibrosis. As fibrosis progresses, the liver surface becomes increasingly irregular, forming nodules and rough edges, leading to increased roughness of the liver’s margin. By calculating the roughness characteristics of the liver’s edge, one can assess the complexity and heterogeneity of the liver surface, which correlates positively with the degree of fibrosis [6].

In recent years, with the advancement of medical imaging technologies [7,8,9,10], such as ultrasound, CT, and MRI, it has become possible to provide information on the morphology, structure, and function of the liver [11]. The feature extraction, classification, and analysis of medical images can achieve non-invasive or minimally invasive qualitative or quantitative assessment of liver fibrosis, offering possibilities for non-invasive or minimally invasive grading of fibrosis [12]. Features in medical images can be broadly categorized into texture features and shape features. Texture features describe the attributes of gray-scale variations and spatial distribution in an image [13], while shape features are quantitative indicators used to describe the morphology of an object [14]. Due to the complexity of individual learners, their performance often fails to meet requirements; ensemble learning can combine multiple weak learners into a strong learner [15]. Boosting, bagging, and stacking are classic algorithms in ensemble learning. Boosting sequentially builds a series of classifiers, adjusting sample weights each round, focusing on incorrectly classified samples to generate multiple prediction functions [16]. Bagging constructs multiple independent learners in parallel, combining their prediction results in the end [17]. Stacking combines the prediction results of multiple base-learning algorithms through a meta-learning algorithm [18]. Stacking ensemble techniques are widely applied; for instance, a stacking ensemble learning framework (SELF) was constructed by Liang M et al. [19] by integrating three machine learning methods, achieving high accuracy in prediction tasks. Cui S et al. proposed a stacking ensemble learning model based on an improved swarm intelligence optimization algorithm, validating its effectiveness on a Chinese earthquake dataset from 1996–2017 [20]. Mota L F M et al. combined stacking ensemble learning with real-time milk analysis to predict cheese production characteristics [21]. Zhang H et al. introduced a multi-dimensional feature fusion and stacking ensemble mechanism (MFFSEM), effectively detecting abnormal network traffic behaviors, achieving commendable results on two intrusion detection evaluation datasets (UNSW-NB15 and CIC-IDS-2017) [22]. Rashid M et al. introduced a tree-based stacking ensemble technique (SET), which, by further enhancing feature selection techniques, better identified normal and anomalous traffic in networks, compared to other existing IDS models [23]. Kardani N et al. used the Artificial Bee Colony (ABC) optimization algorithm to find the best combination of base classifiers and determine the most suitable meta-classifier from 11 machine learning algorithms. The experiments showed that the improved stacking model significantly enhanced the predictive ability for slope stability [24]. By applying meta-heuristic algorithms, suitable solutions, close to the optimal, can be found in a short time for model optimization. The EVO algorithm has a strong global search capability, allowing it to find global optima in complex optimization problems more effectively. The EVO algorithm tends to have higher search efficiency and better convergence performance compared to traditional optimization algorithms. Accordingly, this study proposes an EVO-MS model optimized by the energy valley algorithm (EVO) [25,26,27,28].

The author adapted the micro-unevenness indicators from industrial applications for detecting the shape characteristics of the liver’s edge, selecting materials with significant deformation, such as silicone models, to replace human liver in preliminary tests. Using the SVM model [29] to analyze liver CT images, the study identified feature parameters with significant impact on classification experiments and trained the EVO-MS ensemble model with these parameters. This research aims to explore the effectiveness and applicability of the EVO-MS-based liver fibrosis grading method, providing a new tool for the diagnosis and monitoring of clinical liver fibrosis.

## 2. Materials and Methods

### 2.1. Dataset

**Figure 1:**
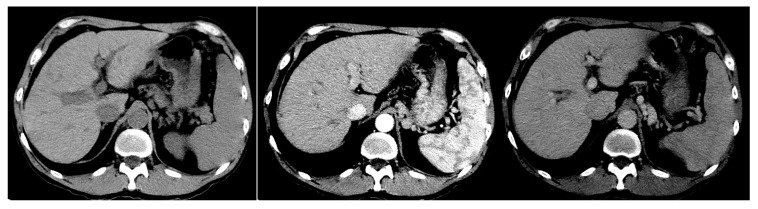
Different CT phased images were obtained from a 52-year-old woman with fibrosis stage F2 due to type C viral hepatitis before and after injection of contrast agent.

All liver CT images in this study were obtained from the Radiology Department of the First Affiliated Hospital of Guangxi Medical University between June 2009 and March 2011 [6,30,31]. The images consist of 415 cases, both diagnosed via liver puncture biopsy and those without a history of liver-related diseases, who did not undergo biopsy. The grading of liver fibrosis was based on the chronic hepatitis fibrosis staging standards revised in 2000 by the Infectious Diseases and Parasitology Branch and the Hepatology Branch of the Chinese Medical Association. The stages were divided into the normal group (S0), the mild fibrosis groups (S1 and S2), the severe fibrosis groups (S3 and S4), and cirrhosis group (CIR), each comprising 70, 69, 69, 69, 69, and 69 cases, respectively. The sample set of imaged CTs included 39 males and 31 females in the normal group, with an average age of 38.60 years; 118 males and 20 females in the mild fibrosis group, with an average age of 37.25 years; 90 males and 48 females in the severe fibrosis group, with an average age of 38.6 years; and 53 males and 16 females in the cirrhosis group, with an average age of 47.5 years. Each image was verified by experienced radiologists to ensure the accuracy of the grading labels. The CT image of the liver is shown in Figure 1.

In practical medical applications, CT scans typically involve the injection of a contrast agent into the patient. The contrast agent, spreading with the blood flow into various tissues and organs, enhances the sensitivity of the tissues to X-rays during scanning. This allows for clearer X-ray signals and better reconstruction of internal body images. The acquired scan images can be categorized according to the timing of the contrast agent injection, as per Table 1. Each scanning phase yields a complete set of full liver cross-sectional images. The CT scanner used was the 64-slice multi-layer spiral CT machine (GE Lightspeed VCT) produced by GE, USA, with an exposure voltage of 120 kV, a tube current of 250 mA, and an image pixel matrix of 512 × 512. The contrast agent used was iohexol injection fluid, administered through an antecubital vein using a high-pressure injector, with a dosage of 85–90 mL, a concentration of 320 mg/mL, and an injection rate of 3.0 mL/s.

### 2.2. Microscopic Roughness

**Figure 2:**
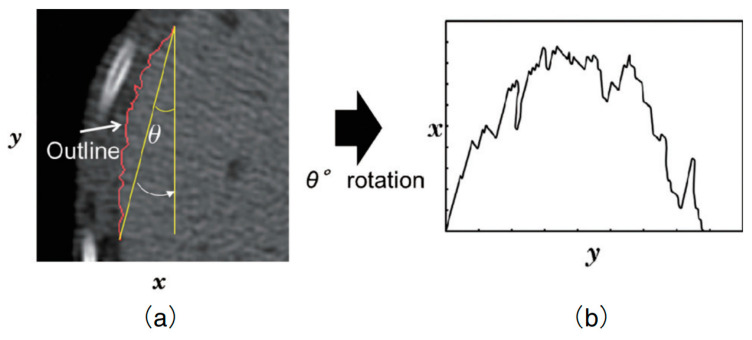
The outline of the hepatic surface, shown in red (**a**), is rotated according to its angle of approximate curve to generate a one-dimensional profile function (**b**).

The hepatic surface profile is outlined with the red line, consisting of more than 128 points, as shown in Figure 2a, on which an approximate curve was determined by a least-square approach, and a one-dimensional function was obtained by drawing a straight line between the start and end points before rotating it parallel to the *y*-axis (Figure 2b). Then, the microscopic roughness of the hepatic surface is calculated as the shape feature.

Micro-unevenness is a quantitative indicator used in mechanical engineering to describe the characteristics of surface morphology. The author intends to select a total of ten such parameters as characteristic parameters, which include l, representing the sampling length, and $Z\left(x\right),$ the profile deviation function.

The average arithmetic deviation of a profile is represented by $R_{a}$. It is the arithmetic mean of the absolute values of the distances. These distances are between the points on the profile line and the baseline. The measurement is taken along the direction of the profile within a sampling length. A smaller $R_{a}$ means a smoother surface. The calculation formula for $R_{a}$ is as follows:

(1) $$R_{a}=\frac{1}{l}\int_{0}^{l}\left|Z\left(x\right)\right|dx$$

The root mean square deviation of the profile is denoted as Rq. It is the square root of the arithmetic mean of the squared distances. These distances are between the points on the profile line and the baseline. Again, the measurement is within a sampling length. A smaller $R_{q}$ value means a smoother surface. The calculation formula for $R_{q}$ is as follows:

(2) $$R_{q}=\sqrt{\frac{1}{l}\int_{0}^{l}Z^{2}\left(x\right)dx}$$

The maximum height of profile micro-unevenness is represented by $R_{max}$. It is the vertical distance between the highest and lowest points on the profile line. This measurement is also within a sampling length. A smaller $R_{max}$ value suggests a smoother surface. The calculation formula for $R_{max}$ is as follows:

(3) $$R_{max}={max}_{0\leq x\leq l}\left|Z\left(x\right)\right|$$

The maximum valley depth of the profile is denoted as $R_{min}$. It is the vertical distance from the lowest point on the profile line to the baseline. This is measured within a sampling length. The calculation formula for $R_{min}$ is as follows:

(4) $$R_{min}={min}_{0\leq x\leq l}\left|Z\left(x\right)\right|$$

The maximum peak height of the profile is denoted as $R_{p}$. It is the highest peak value relative to the mean line within a sampling length. The calculation formula for $R_{p}$ is as follows:

(5) $$R_{p}={max}_{0\leq x\leq l}Z\left(x\right)$$

The average spacing of micro-unevenness of the profile is denoted as $S_{m}$. It is the average distance between the micro-unevenness within a sampling length. The spacing of micro-unevenness refers to the segment length on the mean line between a profile peak and its adjacent valley. Here, n represents the number of profile elements, and $s_{i}$ denotes the width of the ith profile element. A profile element is defined as the segment of the profile line between a peak and its adjacent valley. The calculation formula for $S_{m}$ is as follows:

(6) $$S_{m}=\frac{1}{n}\sum_{i=1}^{n}s_{i}$$

The average spacing of single peaks of the profile is denoted as S. It is the average distance between individual peaks within a sampling length. Here, $x_{i+1}$ represents the position of adjacent peaks, and n is the total number of peaks. The calculation formula for S is as follows:

(7) $$S=\frac{1}{n-1}\sum_{i=1}^{n-1}\left(x_{i+1}-x_{i}\right)$$

The average height of micro-unevenness of the profile is denoted as $R_{z}$. It is calculated as the sum of the average of the five highest peaks and the average of the five deepest valleys within a sampling length. Here, $y_{pi}$ represents the height of the ith highest peak, and $y_{vi}$ denotes the depth of the ith deepest valley. The calculation formula for $R_{z}$ is as follows:

(8) $$R_{z}=\frac{1}{5}\sum_{i=1}^{5}\left(y_{pi}+y_{vi}\right)$$

The density of profile peaks is denoted as D. It is the ratio of the number of profile peaks to the sampling length within a sampling length. Here, n represents the number of profile peaks contained within the sampling length, and l is the sampling length. The calculation formula for D is as follows:

(9) $$D=\frac{n}{l}$$

The profile bearing length ratio is denoted as $t_{p}$. It is the ratio of the bearing length to the sampling length. Given a horizontal intercept, a line parallel to the mean line is drawn at the intercept length below the peaks. The intersection of the profile with this line $l_{1}+l_{2}+l_{2}+\cdots +l_{n}$ is called the bearing length. The calculation formula for $t_{p}$ is as follows:

(10) $$t_{p}=\frac{l_{1}+l_{2}+l_{2}+\cdots +l_{n}}{l}$$

**Figure 3:**
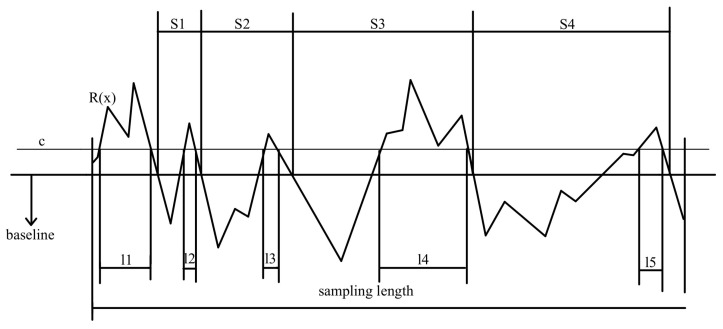
An example of calculating *S_m_* and *t_p_* on the profile.

Examples of $S_{m}$ and $t_{p}$ are illustrated in Figure 3.

### 2.3. Overview of the Proposed Method

**Figure 4:**
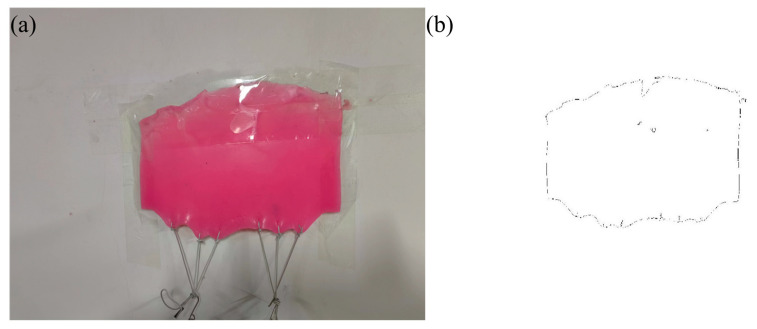
The silicone mold is hung by different weights to simulate the restraint force on the liver caused by the progression of fibrosis (**a**), as shown in its profile image (**b**).

In cases where liver imaging resources were scarce, the authors employed a silicone mold with a favorable degree of deformability as a surrogate for the liver in simulation experiments. Beneath the silicone mold, two sets of holes, totaling six, are uniformly spaced to suspend weights, thereby simulating the edge roughness associated with varying degrees of liver fibrosis. Simulated experiments were conducted to verify the correlation between micro-unevenness parameters and the degree of deformation. The silicone mold and the edges of the silicone are depicted, respectively, in Figure 4.

**Figure 5:**
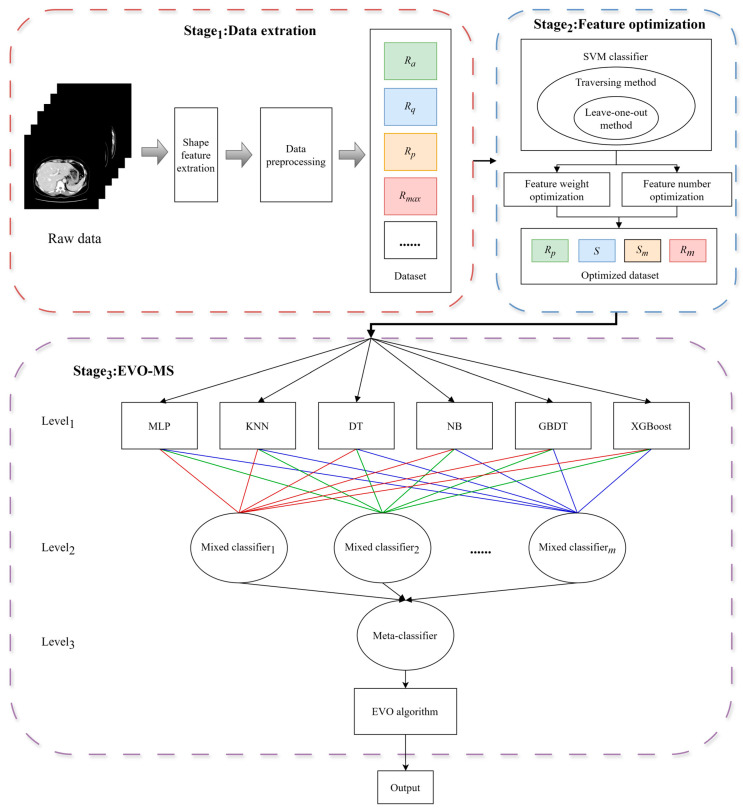
The overall flowchart of staging liver fibrosis based on EVO-MS.

The liver CT experiment comprises three stages: the data extraction module; the feature optimization module; and the EVO-MS classification module. The specific flowchart is illustrated in the Figure 5.

(a) The data extraction module involves the extraction of representative edge curves from the lower segment of the left hepatic lobe to the lower segment of the left hepatic outer lobe on the liver contour map after positioning, rotating, and fitting the edge curve. Based on this edge curve, ten characteristic parameters are extracted. Following min–max normalization of the data, the samples are input into an SVM classifier. The leave-one-out method is employed to maximize the input samples, and an exhaustive search method is used to select different combinations of all feature parameters.

(b) The feature optimization module is divided into two parts: the optimization of the number of feature parameters, and the optimization of the weights of feature parameters. The optimization of the number of feature parameters involves calculating the highest accuracy rate for each feature parameter. The highest accuracy rate for each quantity of feature parameters is denoted as $P\left(k\right)$, where k represents the number of feature parameters selected, and $P\left(2\right)$ represents the highest accuracy rate obtained from $C_{10}^{2}$ combinations when classifying with two feature values. The weight of the feature parameters refers to the degree of influence on the accuracy of the results in the feature parameter SVM classifier. A greater weight indicates a larger impact on accuracy, while a lesser weight suggests a smaller influence on the classification outcome. The frequency of occurrence of ten feature parameters in hierarchical classification combinations is counted.

(11) $$p\left(k\right)=\frac{1}{N}\sum_{i=1}^{N}n_{i}(k)\;\left(k=1,\;2,\;3,\;4,\;5,\;6,\;7,\;8,\;9,\;10,\;i=1,\;2,\ldots ,N\right)$$

(12) $$N=C_{10}^{1}+C_{10}^{2}+C_{10}^{3}+C_{10}^{4}+\cdots +C_{10}^{10}=2^{10}-1$$

where k represents the ten features’ parameter number, $p\left(k\right)$ represents the weight of the k feature parameter. The process iteratively traverses every possible combination of feature parameters to define the classification space using an exhaustive traversal method. The total number of combinations, denoted as N. In this experiment, $n_{i}(k)$ indicates whether the feature quantity k appears in the ith classification space; if the feature quantity appears, $n_{i}(k)=1$, otherwise, $n_{i}(k)=0$. The min–max normalization is employed, where $p{\left(k\right)}_{max}$ is the maximum value of the sample weight data, $p{\left(k\right)}_{min}$ is the minimum value, and the transformation function is as follows:

(13) $$W\left(k\right)=\frac{p\left(k\right)-p{\left(k\right)}_{min}}{p{\left(k\right)}_{max}-p{\left(k\right)}_{min}}$$

(c) After optimizing the number and weights of the feature parameters, those with greater weights were input into the EVO-MS model for training. Initially, the selected six base classifiers—KNN, DT, NB, XGBoost, GBDT, and RF—were utilized to predict the sample and determine the predicted class probabilities, which are denoted as a matrix P:

(14) $$\left.P=\left[\begin{array}{cccc} p_{11} & p_{12} & \cdots & p_{1k}\\ p_{21} & p_{22} & \cdots & p_{2k}\\ \vdots & \vdots & \ddots & \vdots \\ p_{n1} & p_{n2} & \cdots & p_{nk} \end{array}\right.\right]$$

where n denotes the number of base models, k represents the number of splits, and $p_{ij}$ signifies the predicted probability of the ith base model for the jth split. Subsequently, the probability values outputted by the base models are fed as input data into a mixture layer composed of m mixing units. The predictive probabilities from the mixture layer are then input into the meta-model layer. A logistic regression method is employed to synthesize the predictions of the various mixed classifiers, thereby yielding a more accurate final forecast. The hyperparameter combinations of each base model and the weights of the individual units in the mixture layer are optimized using the energy valley optimization algorithm, with the optimization process utilizing cross-entropy loss as the objective function.

### 2.4. Multiple Model

**Figure 6:**
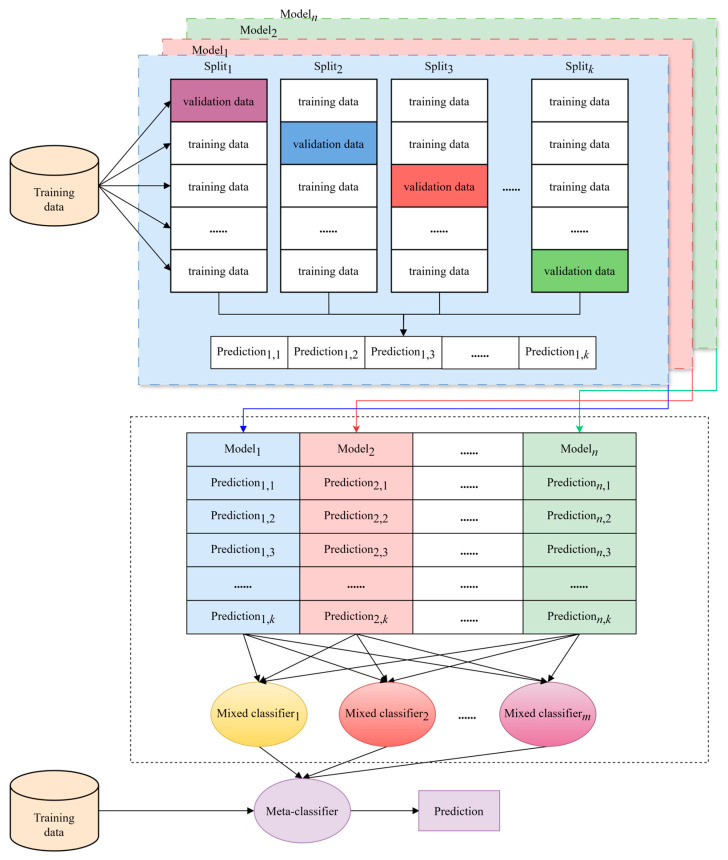
The architecture diagram of the multiple stacking algorithm.

The multiple stacking model is an ensemble learning algorithm that enhances the efficiency of complex data processing by integrating the predictive capabilities of various base models. The multiple stacking architecture comprises three levels: the base models, the blending layer, and the meta-model. The base models include a diverse array of machine learning algorithms, which are independently trained on data using K-fold cross-validation to ensure the model’s generalizability. The output of the base model layer, consisting of the prediction results of each model on the data, is fed into the blending layer. In this layer, the predictions of the base models are used to train multiple ensemble models responsible for learning how to most effectively combine the predictions of the base models. The output of the blending layer is then used as the input for the meta-model, which further optimizes the prediction results to achieve higher accuracy than individual models. The advantage of the multiple stacking model lies in its ability to capture the complementary information between different models. By learning the differences in predictions of various models, it enhances the overall predictive performance and improves the model’s generalization ability on unknown data. The multiple stacking algorithm is illustrated in Figure 6, where k denotes the use of *k*-fold cross-validation, and ${Prediction}_{i,j}$ denotes the predicted probability of the ith model on the jth split.

### 2.5. Energy Valley Optimization

The energy valley optimizer (EVO) is a metaheuristic algorithm grounded in physical principles, inspired by the stability and decay processes of particles. In the cosmos, the majority of particles are considered unstable, with only a select few capable of maintaining permanence. Unstable particles release energy through decay, with the decay rate varying slightly among different particle types. The energy valley focuses on particle stability, determined by the binding energy of particles and their interactions with others. Depending on the stability level of the particles, each tends to increase its stability level by adjusting the ratio of neutrons to protons and moving towards the stability band or the bottom of the energy valley. During the decay process, a particle with a lower energy level is produced, while excess energy is emitted. The decay processes of particles with different stability levels yield three types of emissions, corresponding to three position update processes. Two of these processes occur within the decision variables, executing the exploration process, while one position update process occurs within the candidate solutions, satisfying exploitation. These principles provide the foundation for the EVO algorithm, enabling it to optimize the performance of solutions by simulating the stability and decay processes of particles.

The initial step of the EVO algorithm is initialization, where particles (candidate solutions) $X_{i}$ within the search space are established, representing various levels of stability. Assuming the search space is a specified section, a random initialization operation is conducted:

(15) $$X_{i}=\left[\begin{array}{c} x_{1}^{1}\cdots x_{1}^{j}\cdots x_{1}^{d}\\ ...\vdots \\ ...\vdots \\ x_{i}^{1}\cdots x_{i}^{j}\cdots x_{i}^{d}\\ ...\vdots \\ ...\vdots \\ x_{n}^{1}\cdots x_{n}^{j}\cdots x_{n}^{d} \end{array}\right],\;\left\{\begin{array}{c} i=1,2,\ldots ,n.\\ j=1,2,\ldots ,d. \end{array}\right.$$

(16) $$\left.x_{i}^{j}=x_{i,min}^{j}+rand\left(x_{i,max}^{j}-x_{i,min}^{j}\right),\;\left\{\begin{array}{l} i=1,2,\ldots ,n.\\ j=1,2,\ldots ,d. \end{array}\right.\right.$$

where n represents the total number of particles, d denotes the dimensions of the problem under consideration, $x_{i}^{j}$ signifies the jth decision variable of the initial position of the ith particle, while $x_{i,min}^{j}$ and $x_{i,max}^{j}$, respectively, represent the lower and upper bounds of the jth decision variable within the ith particle; rand is a random number uniformly distributed in the interval [0, 1].

The second step of the energy valley algorithm involves determining the enrichment boundary (EB) for the particles. Each particle is assessed through the objective function, establishing its neutron enrichment level ($NEL_{i}$), which is utilized to distinguish between neutron-poor and neutron-rich particles.

(17) $$EB=\frac{\sum_{i=1}^{n}NEL_{i}}{n},i=1,2,\ldots ,n.$$

where $NEL_{i}$ represents the neutron enrichment energy level of the ith particle, while EB denotes the enrichment boundary for particles in the universe.

The third step of the energy valley algorithm is to evaluate the stability level of the particles, based on the objective function:

(18) $$SL_{i}=\frac{NEL_{i}-BS}{WS-BS},i=1,2,\ldots ,n.$$

where $SL_{i}$ denotes the stability level of the ith particle, while BS and WS represent the particles with the best and worst stability levels within the universe, respectively. Their stability levels are determined by the minimum and maximum values of the objective function.

Within the main search loop of the energy valley optimization (EVO), if the neutron enrichment level of a particle exceeds the enrichment threshold ($NEL_{i}>EB$), it is postulated that the particle possesses a higher neutron-to-proton ratio. Depending on the stability level of the particle, three decay processes (α, β, γ) are adopted accordingly. To simulate the stability boundary (SB) in the cosmos, a random number within the interval [0, 1] is generated. Should the stability level of the particle surpass the stability boundary (${SL}_{i}>EB$), α and γ decays may occur, as these decays are pertinent for heavy particles with higher stability. In accordance with the physical principles of α decay, the emission of α rays facilitates the enhancement of the stability of the reaction products. This process serves as one of the EVO position update mechanisms, thereby generating new candidate solutions. Specifically, two random integers, Alpha Index I, are generated within the interval of [1,d] to represent the quantity of emitted α rays. Subsequently, within the [1,Alpha Index I] interval, a value for Alpha Index II is determined to specify the particular α rays to be emitted. The emitted rays, being decision variables within the candidate solution, are removed and replaced by the rays from the particle with the highest level of stability ($X_{BS}$) or from the α rays within the candidate solution. The pertinent mathematical formulas are as follows:

(19) $$\left.X_{i}^{New1}=X_{i}(X_{BS}(x_{i}^{j})),\left\{\begin{array}{c} i=1,2,\ldots ,n.\\ j=Alpha\;Index\;II. \end{array}\right.\right.$$

where a new particle is generated, denoted as $X_{i}^{New1}$, while $X_{i}$ represents the current position vector of the ith particle (solution candidate) within the universe (search space). The position vector of the particle with the optimal stability level is denoted as $X_{BS}$, and $x_{i}^{j}$ represents the jth decision variable or emitted rays. Moreover, in the gamma decay process, γ rays are emitted to elevate the stability level of the excited particles. This process can act as another position update mechanism for EVO, generating new candidate solutions in the process. For this purpose, within the interval [1,d], two random integers, referred to as Gamma Index I, are generated to represent the number of γ rays to be emitted. A value for Gamma Index II is determined within the interval [1,Gamma Index I] to specify the γ rays to be considered within the particle. The γ rays within the particle, serving as decision variables in the candidate solution, are removed and replaced by those from adjacent particles or candidate solutions ($X_{Ng}$), emulating the interaction of the excited particles with other particles or even magnetic fields. The total distance between the considered particle and other particles is calculated as follows:

(20) $$\left.D_{i}^{k}=\sqrt{{\left(x_{i}-x_{k}\right)}^{2}+{\left(y_{i}-y_{k}\right)}^{2}},\left\{\begin{array}{c} i=1,2,\ldots ,n.\\ k=1,2,\ldots ,n-1. \end{array}\right.\right.$$

where $D_{i}^{k}$ represents the total distance between the ith particle and the kth adjacent particle, while $\left(x_{i},y_{i}\right)$ and $\left(x_{k},y_{k}\right)$ denote the coordinates of the particle in the search space. When considering the *i*th particle, compute its position relative to the other *n* − 1 particles and identify the nearest *k*th particle. Utilizing these operations, the position update process for generating the second candidate solution is as follows:

(21) $$\left.X_{i}^{New2}=X_{i}(X_{Ng}(x_{i}^{j})),\left\{\begin{array}{c} i=1,2,\ldots ,n.\\ j=Gamma\;Index\;II. \end{array}\right.\right.$$

where a new particle, denoted as $X_{i}^{New2}$, is generated, and $X_{i}$ represents the current position vector of the ith particle (solution candidate) within the cosmos (search space). Additionally, $X_{Ng}$ denotes the position vector of the neighboring particles surrounding the ith particle, and $x_{i}^{j}$ represents the jth decision variable or emitted photons. If the stability level of the particle falls below the stability threshold (${SL}_{i}\leq EB$), β decay is presumed to have occurred, as such decay processes occur in unstable particles with lower stability. In accordance with the physical principles of β decay, particles emit β rays to enhance their stability level; hence, those particles with higher levels of instability should perform larger jumps within the search space. During the position update process, particles move towards the optimal stability level ($X_{BS}$) and the particle center ($X_{CP}$). This simulates the behavior of particles gravitating towards the stability band, where most known particles congregate, typically exhibiting higher stability. The relevant mathematical formulas are as follows:

(22) $$X_{CP}=\frac{\sum_{i=1}^{n}X_{i}}{n},i=1,2,\ldots ,n.$$

(23) $$X_{i}^{New1}=X_{i}+\frac{\left(r_{1}\times X_{BS}-r_{2}\times X_{CP}\right)}{SL_{i}},i=1,2,\ldots ,n.$$

where $X_{i}^{New1}$ and $X_{i}$, respectively, represent the future and current position vectors of the ith particle (solution candidate) within the universe (search space). $X_{BS}$ denotes the position vector of the particle with the optimal stability level, while $X_{CP}$ is the position vector of the particle center. $SL_{i}$ is the stability level of the ith particle, and $r_{1}$ and $r_{2}$ are two random numbers within the interval [0, 1], determining the amplitude of the particle’s motion. To enhance the development and exploration level of the algorithm, a new position update mechanism is implemented for particles with β decaying stability level. This mechanism, without affecting the particle’s motion, is achieved by controlling the movement of the particle with the highest level of stability ($X_{BS}$), as well as the movement of adjacent particles or candidate particles ($X_{Ng}$). The mathematical formula is as follows:

(24) $$X_{i}^{New2}=X_{i}+\left(\begin{array}{c} r_{3}\times X_{BS}-r_{4}\times X_{Ng} \end{array}\right),i=1,2,\ldots ,n.$$

where $X_{i}^{New2}$ and $X_{i}$ represent the future and current position vectors of the ith particle (solution candidate) in the universe (search space), respectively. $X_{BS}$ is the position vector of the particle with the optimal stability level, and $X_{BS}$ is the position vector of the neighboring particles around the ith particle; $r_{3}$ and $r_{4}$ are two random numbers within the [0, 1] interval that determine the amount of particle movement. If the neutron enrichment level of a particle is below the enrichment threshold ($NEL_{i}\leq EB$), it is considered that the particle has a relatively small proton-to-neutron ratio, and the particle is more inclined to migrate towards the stability band through processes such as electron capture or positron emission. The random motion in the search space is characterized as the following types of movement:

(25) $$X_{i}^{New}=X_{i}+r,i=1,2,\ldots ,n.$$

where $X_{i}^{New}$ and $X_{i}$ represent the future and current position vectors of the ith particle (solution candidate) in the universe (search space), and r is a random number within the [0, 1] interval that determines the magnitude of the particle’s movement.

At the end of the EVO main loop, if a particle’s enrichment level is above the enrichment threshold, each particle generates only two new position vectors, $X_{i}^{New1}$ and $X_{i}^{New2}$, while for particles with lower enrichment levels, only $X_{i}^{New}$ is generated as the new position vector. In each state, the newly generated vectors are merged with the current population, and the best particle participates in the next search cycle of the algorithm. For decision variables that exceed the predefined upper and lower bounds, a boundary violation flag is determined, and the maximum number of evaluations of the objective function or the maximum number of iterations is used as the termination criterion. The pseudo-code of the energy valley optimization algorithm is presented in the Table 2.

**Figure 7:**
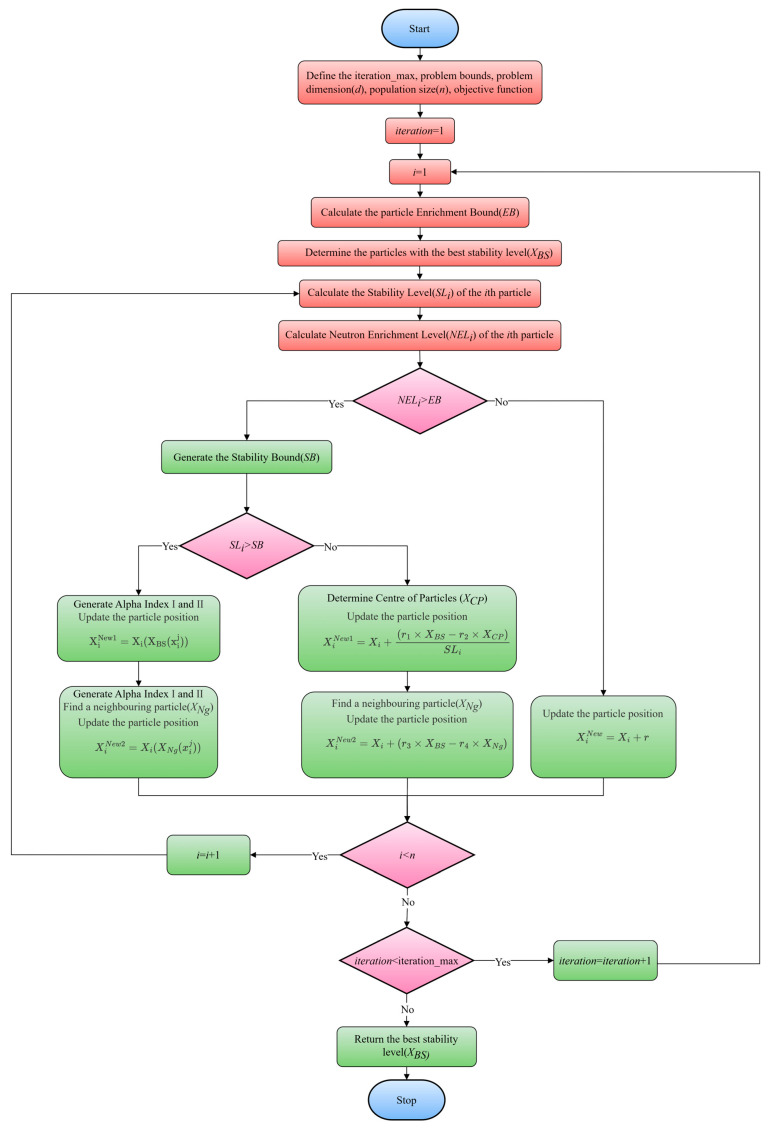
The flow diagram of the energy valley optimization algorithm.

The flow diagram of the energy valley optimization algorithm is shown in Figure 7.

## 3. Results

### 3.1. Feature Optimization

**Figure 8:**
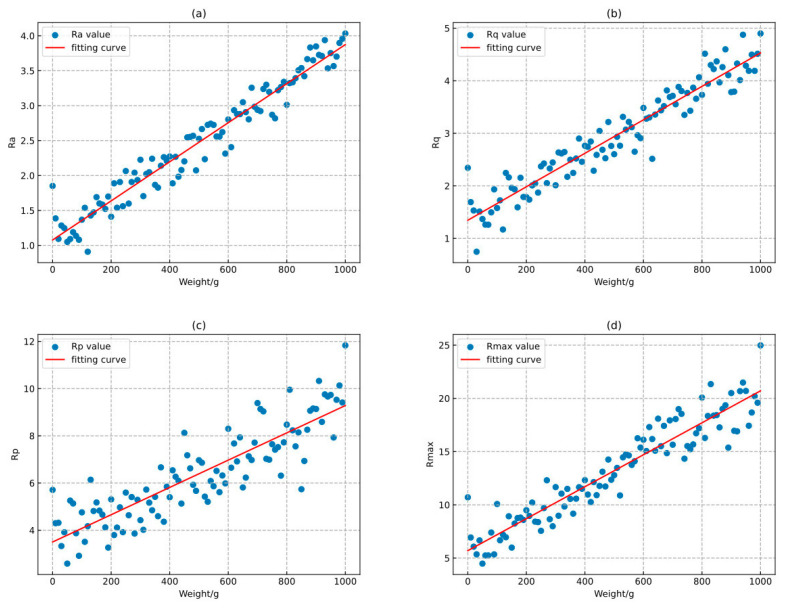
Feature parameter-quality curve. (**a**) Ra-quality. (**b**) Rq-quality. (**c**) Rp-quality. (**d**) Rmax-quality.

In the feature extraction phase, a silicone simulation experiment was initially conducted to present the relationship between representative feature parameters $R_{a}$, $R_{q}$, $R_{p}$, $R_{max}$ and the mass of the weights. As illustrated in Figure 8a–d, with the gradual increase in the mass of the weights, the values of these feature parameters also correspondingly rise, indicating a significant positive correlation between them. This outcome confirms that the feature parameters can effectively reflect the changes in the mass of the weights. The blue data points in Figure 8 represent the feature parameter values measured at different weight masses, while the red fitting line is obtained through least squares fitting, providing us with the best estimate of the trend within the dataset.

**Figure 9:**
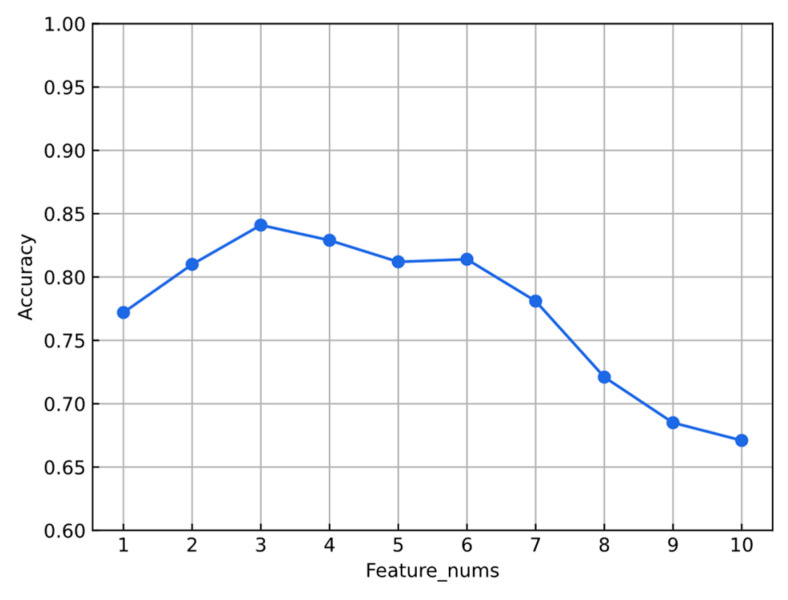
Optimization of the number of feature parameters.

In the liver CT experiments, the quantity of feature parameters was optimized using an SVM classifier. The statistical analysis of the experimental results for the highest accuracy rates of shape feature quantities at various counts reveals that the classifier achieves superior classification performance when the number of feature parameters ranges from three to five. A moderate number of feature parameters aids in enhancing the classifier’s accuracy and efficiency. In contrast, an excess of feature parameters was observed to negatively impact the classifier’s performance due to data redundancy, leading to a decrease in classification accuracy. Conversely, when the number of feature parameters is insufficient, the classifier is unable to effectively distinguish between different categories due to a lack of adequate information. The optimization of the number of feature parameters results is depicted in the Figure 9.

**Figure 10:**
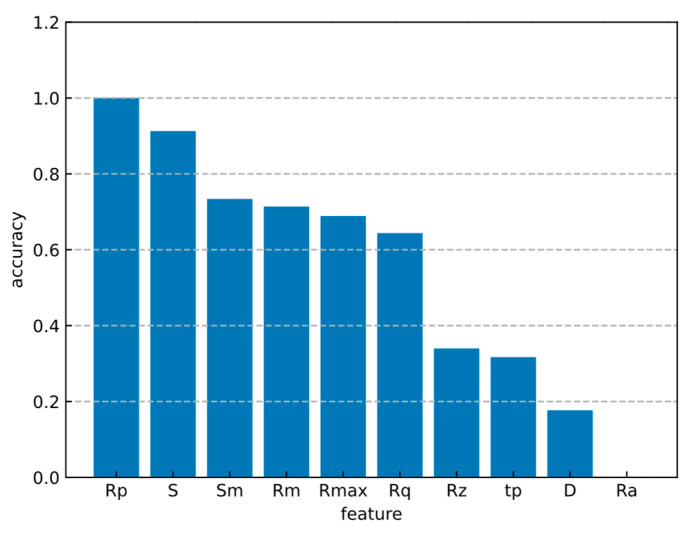
Weight of feature parameters.

During the optimization of feature parameter weights, the experimental results of feature parameter weight optimization are displayed in descending order, as shown in Figure 10. The results demonstrate that the weights of five feature parameters—$R_{p}$, S, $S_{m}$, $R_{min}$, and $R_{max}$—significantly influence the accuracy of the classification. These parameters are highly relevant in the diagnosis of liver fibrosis, providing an accurate reflection of the degree of liver fibrosis pathology. In the field of mechanical engineering, measurements of micro-surface unevenness that are widely recognized as representative include the maximum height of $R_{max}$, $R_{p}$, $R_{min}$. This confirms the theoretical basis for applying these micro-unevenness quantification indicators to the medical domain, utilizing them to detect the edge roughness associated with the degree of liver fibrosis, and serving as a criterion for grading.

### 3.2. EVO-MS Model Performance

The dataset for this study encompasses a total of 415 cases, including both patients diagnosed with liver fibrosis via liver biopsy and individuals without a history of liver-related diseases, examined at the First Affiliated Hospital of Guangxi from June 2009 to March 2011. The data were divided into a training set and a test set at a ratio of 7:3. To validate the effectiveness of the model, its performance was assessed on the test set using various evaluation metrics, including the construction of Receiver Operating Characteristic (ROC) curves and calculation of AUC, accuracy, precision, sensitivity, specificity, and F1-score. Moreover, the Wilcoxon signed-rank test is employed to compare the EVO-MS model with the other six models.

**Figure 11:**
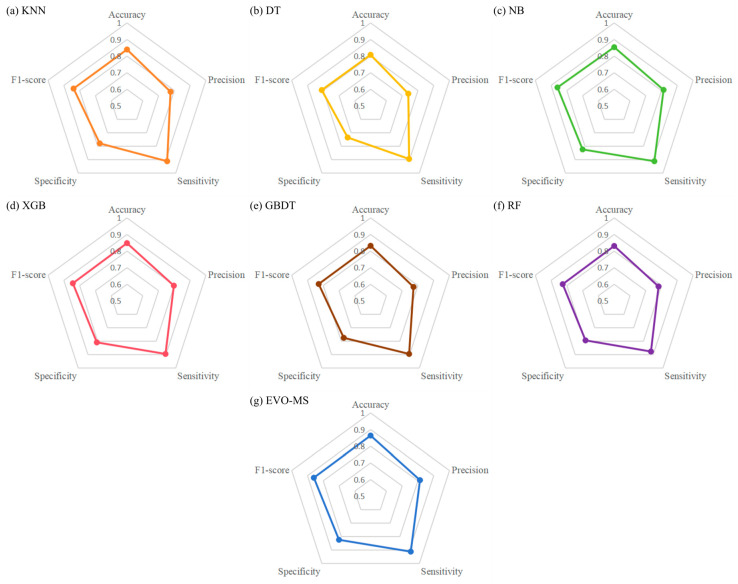
The scores of each model on the five metrics: (**a**) KNN. (**b**) DT. (**c**) NB. (**d**) XGB. (**e**) GBDT. (**f**) RF. (**g**) EVO-MS.

The EVO-MS model and the six individual models’ performance metrics at a prediction threshold of 0.5, such as the validation set’s root mean square error (RMSE), test set RMSE, accuracy, precision, sensitivity, and specificity, are shown in the Table 3. It is evident from the Table 3 that our proposed EVO-MS outperforms the other six individual models, achieving the highest levels among the seven models, with an accuracy of 0.864, a precision of 0.813, a sensitivity of 0.912, a specificity of 0.824, and an F1-score of 0.860. The EVO-MS model’s accuracy, precision, sensitivity, specificity, and F1-score are, respectively, 5.6%, 7.4%, 3.5%, 8.9%, and 5% higher than those of the lowest-scoring model. The scores of each model on the five metrics are illustrated in the Figure 11. The results of the Wilcoxon signed-rank test, as delineated in Table 4, reveal significant variances between the EVO-MS model and the other six models (p < 0.05). Considering evaluation metrics such as AUC, sensitivity, and specificity, it is inferred that the overall performance of the EVO-MS model is superior to that of the competing models.

**Figure 12:**
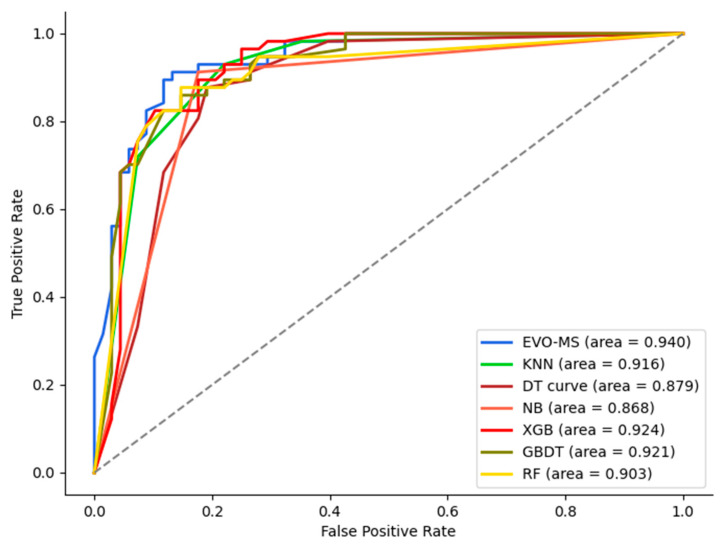
The ROC curves for each model.

The Area Under the Curve (AUC) values for the models EVO-MS, KNN, DT, NB, XGB, GB, and RF are 0.940, 0.916, 0.879, 0.868, 0.924, 0.921, and 0.903, respectively. The proposed EVO-MS model achieved the highest AUC. The ROC (Receiver Operating Characteristic) curves for each model are illustrated in the Figure 12 below:

## 4. Discussion

The diagnosis and grading of liver fibrosis is a critical fundamental task. This study has constructed a multiple stacking model, optimized by energy valley optimization (EVO), based on 415 CT images from different stages of liver fibrosis. By analyzing and extracting shape features, accurate grading of the extent of liver fibrosis can be achieved, which has the potential to change the current reliance on invasive tests, such as liver biopsies.

The statistical results indicate that selecting three shape features yields better classification performance, with the maximum peak height of the contour, the average inter-peak distance of the contour, and the average inter-roughness distance of the contour microstructure having significant weights. In terms of model construction, the EVO-MS combines six individual base models (KNN, DT, NB, XGBoost, GBDT, and RF). For parameter tuning, we employ the EVO algorithm to replace the manual selection process. The EVO-MS model demonstrates excellent performance in grading liver fibrosis on the test set, achieving commendable scores across the five main evaluation metrics (an accuracy of 0.864, a precision of 0.8125, a sensitivity of 0.9123, a specificity of 0.8235, and an F1-score of 0.8595), outperforming the lowest-scoring model by 3.2%, 4%, 1.7%, 4.5%, and 3.1%, respectively. This indicates that the EVO-MS model can more effectively detect different stages of liver fibrosis.

Our work is primarily limited by the size of the dataset. Due to patient privacy protection, high data annotation costs, and the dispersed, non-shared nature of the data, it is challenging to obtain large-scale liver fibrosis CT image datasets. With more abundant data in the future, we hope to further improve the model’s performance. Moreover, after further comparison with clinical presentations by doctors, we anticipate providing a new tool for the grading of liver fibrosis.

## Figures and Tables

**Table 1:** The scanning time for each phase on contrast CT images.

Scan Phase	CT Scan Timing	Contrast Agent Diffusion
N Phase: Non-contrast Phase	<0 s	No contrast agent injected
A Phase: Arterial Phase	25 s	Contrast agent diffused into hepatic arterial vessels
V Phase: Venous Phase	60 s	Contrast agent refluxed into hepatic venous vessels
P Phase: Equilibrium Phase	120 s	Contrast agent diffused into hepatic capillary tissues

**Table 2:** The pseudo-code of the energy valley optimization algorithm.

EVO Pseudo-Code
*Define the iteration_max, problem bounds, problem dimension (*d*), population size* (n*),* *objective function.* *Calculate the fitness values of all candidate particles based on the neutron enrichment level (*${NEL}_{i}$*)* *while iteration < iteration_max do* *Calculate the particle enrichment boundary (*EB*)* *Determine the particle with the best stability level (*$X_{BS}$*)* *for* i=1:n *do* *Calculate the stability level (*${SL}_{i}$*) of the* ith *particle* *Calculate the neutron enrichment level (*${NEL}_{i}$*) of the* ith *particle* *if* ${NEL}_{i}>EB,$ *then* *Generate the stability bound (*SB*)* *if* ${SL}_{i}>SB,$ *then* *Generate* Alpha Index I and II *for* j=1:Alpha Index I and II *do* $X_{i}^{New1}=X_{i}(X_{BS}(x_{i}^{j}))$ *end* *Generate* Gamma I and II Find a neighboring particle ($X_{Ng}$) *for* j=1:Gamma II *do* $X_{i}^{New2}=X_{i}(X_{Ng}(x_{i}^{j}))$ *end* *else if* ${SL}_{i}\leq SB$ *then* *Determine center of particles (*$X_{CP}$*)* $X_{i}^{New1}=X_{i}+\frac{\left(r_{1}\times X_{BS}-r_{2}\times X_{CP}\right)}{SL_{i}}$ *Find a neighboring particle (*$X_{Ng}$*)* $X_{i}^{New2}=X_{i}+\left(\begin{array}{c} r_{3}\times X_{BS}-r_{4}\times X_{Ng} \end{array}\right)$ *end* *else if* ${NEL}_{i}\leq EB$ *then* $X_{i}^{New}=X_{i}+r$ *end* i=i + 1 *end* *iteration = iteration + 1* *end* *Return the best stability level (*$X_{BS}$*)*

**Table 3:** The scores of each model across five metrics.

	Accuracy	Precision	Sensitivity	Specificity	F1-Score
EVO-MS	0.864	0.813	0.912	0.824	0.860
KNN	0.840	0.776	0.912	0.779	0.839
DT	0.808	0.739	0.895	0.735	0.810
NB	0.854	0.813	0.912	0.824	0.860
XGB	0.848	0.797	0.895	0.809	0.843
GBDT	0.832	0.772	0.895	0.775	0.829
RF	0.831	0.781	0.877	0.794	0.826

**Table 4:** The Wilcoxon signed-rank test between the EVO-MS model and the other models.

Pair Comparison	p Value
EVO-MS vs. KNN	0.041
EVO-MS vs. DT	0.040
EVO-MS vs. NB	0.039
EVO-MS vs. XGB	0.000
EVO-MS vs. GBDT	0.045
EVO-MS vs. RF	0.037

## Data Availability

The real medical datasets were obtained from the Radiology Department of the First Affiliated Hospital of Guangxi Medical University. The dataset is available on reasonable requests from the corresponding author.
